# Annotations

**Published:** 1895-04-27

**Authors:** 


### Annotations.

### The Doctor on Guard.

Every day of the year, Sundays excepted, the medical officer for the Port of London examines critically on the average about 100 separate individuals who may be the bearers of infectious diseases into our vast city. What seems to be still more striking is that he inspects in detail every year, or is responsible for the inspection of nearly 27,000 separate ships. During the six months ending December 31st, 1894, he inspected 13,471 seagoing vessels. It may be noted, by the way, that of those 13,471 as many as 81 per cent. were the property of British owners. It is also incidentally mentioned that about half of these were vessels trading to foreign ports and the other half coasters. If it be asked, what is the use of all this careful examination, the reply is that during last year as many as 62 persons were discovered and isolated who were the victims of infectious diseases; and every one of those persons—more than one for every week in the year—might, if not isolated, have become a centre from which infectious disease might have spread throughout London and the whole country. How much of our most happy freedom from epidemics we owe to the watchfulness of our port medical officers we do not know, and we never can know. Alien immigrants, often brought to our ports sickening from their own filthiness, were examined to the number of 3,425 last year. It is hardly too much to say that every one of those 3,425 immigrants was a "dangerous person." In point of fact the work done at the Port of London under Dr. Collingridge's authority is of a sanitary value which is simply priceless to the community.

### An Ambulance System for Paris.

Ambulance work is one of those things which they do not "do better in Paris." Up to the present time, indeed, the capital of the great French Republic has not furnished her citizens with any complete ambulance system at all. But, if we may depend upon the assurances of M. Marcel Baudouin, the Paris authorities are minded to promptly change all that. M. Baudouin, in a lengthy communication, tells his fellow citizens of the wonders which "first aid" has achieved in Chicago and other American towns, and notes with a generosity truly French, that "even in London" some ambulance work is being done. Perhaps if M. Baudouin would condescend to pay us a visit and inspect our London system, noting especially its simplicity, economy and effectiveness, he might be inclined to give us a little less grudging praise? Be that as it may, he is quite resolved that an ambulance system shall be established in Paris; and that it shall be as compact, complete, and logical in its details as an essay by a French philosopher. All Paris is to be mapped out into more or less equal districts; an ambulance station is to be placed in each district; notice-boards are to be put up at every conspicuous corner indicating the nearest "first aid" station; near each notice-board is to be found a telephone, by means of which the nearest ambulance station can be immediately communicated with, and from which an ambulance, and, if need be, a doctor can be dispatched to the scene of accident. Every station will be in telephonic communication with the central hospital for the district; and thus the Parisian who is unfortunate enough to break his leg or his head in the street will be tucked up in bed in a Paris hospital almost before one can draw a second breath. The system, on paper, is admirable. Let us hope that Paris and M. Marcel Baudouin will see it converted into an accomplished fact without an hour's unnecessary delay.

### "Biliousness."

It is impossible to alter the English language for the sake of ensuring pathological accuracy, and however erroneous may be the idea conveyed, the phrase, "bilious attack," stands as representing a group of symptoms which are popularly supposed to point to disordered liver as their origin. It is important, however, not to be misled by such phraseology. Confident as the man in the street may be in the efficacy of his liver as the cause of all his woes, we know, and ought to act upon the knowledge, that in so-called "biliousness" or "bilious attacks" the liver is only a secondary sufferer. In a lecture by Dr. Saundby, recently published in the *Clinical Journal*, it is emphatically asserted that an ordinary bilious attack is really an attack of catarrhal inflammation of the stomach and duodenum. Perhaps we should feel inclined to lay more emphasis than he does on the importance of the neurotic element and the necessity, and even the difficulty, of differentiating from pure gastro-duodenal catarrh, cases of migraine or of gastric neuralgia. It is clear enough to those who see much of such cases that no strict line of demarcation can be always drawn. It thus often enough occurs that, even in obviously neurotic ailments, the traditional blue pill and saline are found of service; not that they cure the neurosis, but that they relieve the super-added inflammation. Still it must be recognised that "biliousness" and "bilious attacks" are related to stomach and duodenum, and not to liver, and that in their causation there is an inflammatory element which must be considered in their treatment. If we look on "biliousness" as due to a "torpid liver" which requires stimulating, we land ourselves in the fogs of some of the most disputed points in therapeutics; but if we accept what physiological pathology teaches, viz., that it is the result of an inflammatory change in the mucous lining of the stomach and duodenum, the treatment becomes plain. Simple unirritating food, which will not injure the tender lining of the stomach, and will not by its fermentation stretch and strain it, together with such aperients as will drain it speedily of its contents and leave it soon at rest, give us the best hope of quick relief. Dr. Saundby says that Abernethy defined a bilious attack as that condition which is relieved by blue pill, and it is still true that five or ten grains of blue pill at bedtime, followed in the morning by a teaspoonful of Carlsbad salts dissolved in hot water, and sipped while dressing, gives the best results in this complaint. The treatment of a bilious attack is then to be conducted not on vague notions of stimulating the liver, but in accordance with the well-known principles of treating inflammation wherever it may arise—rest so far as may be to the inflamed part, and drainage (by blue pill) to remove the products of its inflammation.

